# Discrepancies between Fundus Photography and Multimodal Imaging in Mapping of Choroidal Tumor Borders

**DOI:** 10.1016/j.xops.2025.101057

**Published:** 2025-12-30

**Authors:** Darvy Dang, Meghna Burmi, Xavier Hadoux, Daniel McKay, Maxime Jannaud, Myra B. McGuinness, Peter van Wijngaarden, Roderick O’Day

**Affiliations:** 1 Centre for Eye Research Australia, Royal Victorian Eye and Ear Hospital, Melbourne, Victoria, Australia; 2 Ocular Oncology Unit, Royal Victorian Eye and Ear Hospital, Melbourne, Victoria, Australia; 3 Faculty of Medicine, Nursing and Health Sciences, Monash University, Melbourne, Victoria, Australia; 4 Department of Surgery (Ophthalmology), University of Melbourne, Melbourne, Victoria, Australia; 5 The Florey Institute of Neuroscience and Mental Health, Melbourne, Victoria, Australia

**Keywords:** Choroidal melanoma, Choroidal nevus, Choroidal tumor, Multimodal imaging, Enhanced depth imaging optical coherence tomography

## Abstract

**Purpose:**

Accurate choroidal tumor border mapping is required for their management. We compared border mapping accuracy between unimodal assessment (color fundus photography [CFP] or scanning laser ophthalmoscopy [SLO]) against a multimodal assessment (CFP, SLO, and OCT) and identified tumor characteristics that affect performance.

**Design:**

A cross-sectional diagnostic accuracy study.

**Participants:**

Sixty-four choroidal lesions (61% nevi, 39% melanomas; median basal diameter 5.65 mm, median thickness 1.85 mm) from 63 patients at tertiary ocular oncology clinics in Victoria, Australia. No separate control group was included.

**Methods:**

Two ocular oncologists independently delineated lesion margins on CFP and SLO. Multimodal assessment was established by agreement. Agreement between unimodal and multimodal assessments was quantified using the 95th percentile Hausdorff Distance (HD95).

**Main Outcome Measures:**

The HD95 in millimeters between unimodal and multimodal tumor borders. Dice coefficient summary statistics are also provided.

**Results:**

Overall, unimodal CFP and SLO assessments had good agreement with multimodal assessments (median HD95 <1 mm for each grader and device). However, HD95 was >2 mm in 5% (grader 1) and 9% (grader 2) of CFP assessments and in 2% (grader 1) and 3% (grader 2) of SLO assessments. Nonpigmented and mixed-pigmented tumors showed significantly higher HD95 than pigmented lesions for most grader-modality pairs, particularly for grader 1 on CFP and SLO (*P* < 0.05).

**Conclusions:**

Choroidal tumor margin assessment was accurate on CFP and SLO as compared with a multimodal assessment that included enhanced-depth imaging OCT (EDI-OCT). However, the borders of a subset of tumors, especially those with reduced pigmentation, were inaccurately determined when using fundus photography alone. Incorporating EDI-OCT into choroidal tumor border mapping may reduce these discrepancies.

**Financial Disclosure(s):**

Proprietary or commercial disclosure may be found in the Footnotes and Disclosures at the end of this article.

Knowing the location of the edge of a choroidal tumor is a prerequisite to its management. It enables an assessment of size, the ability to monitor for growth, and the accurate placement of therapy. Clinicians typically perform choroidal tumor margin assessment ophthalmoscopically or by fundus photography.^1^ This relies on the presence of contrast between the tumor tissue and the surrounding choroid and overlying retina. If there is a lack of contrast, for example because the color of the tumor matches the surrounding tissue, or there are pathological changes to the overlying ocular tissues, it is plausible that this assessment may be inaccurate.

The use of multimodal imaging in ocular oncology is standard of care. It can be used to identify the factors that predict whether a choroidal naevus will undergo malignant growth^1^ and to define the imaging features of choroidal metastases^2^ and vitreoretinal lymphoma.^3^ Each of the imaging technologies obtains different information from different locations in the eye. Enhanced-depth imaging OCT (EDI-OCT) collects cross-sectional information of the choroid, and melanocytic choroidal tumors have distinctive features enabling it to be distinguished from the normal choroid.^4^^,^^5^ Incorporating EDI-OCT into choroidal tumor margin assessment may assist when there is a lack of contrast or abnormalities of the overlying tissues, but the cross-sectional EDI-OCT images need to be transposed into an *en face* image, and the scanning pattern needs to be dense.

In this study, we aimed to investigate the accuracy of choroidal tumor margin assessment using the most commonly used fundus photography—color fundus photography (CFP) and scanning laser ophthalmoscopy (SLO)—as compared to a multimodal assessment that included EDI-OCT.

## Methods

This cross-sectional diagnostic accuracy study was approved by the Royal Victorian Eye and Ear Hospital Human Research Ethics Committee and adhered to the tenets of the Declaration of Helsinki. Informed consent was obtained from each participant. Consecutive patients who met the inclusion criteria were enrolled and imaged between March 2021 and October 2023 from 3 ocular oncology clinics in Victoria, Australia.

### Study Participants

Inclusion criteria were age ≥18 years and a diagnosis of choroidal nevus or choroidal melanoma by an ocular oncologist. Lesions were classified as choroidal nevi or small melanomas based on standard clinical criteria including lesion thickness, basal diameter, presence of subretinal fluid, orange pigment, documented growth, and ancillary imaging findings (OCT and ultrasonography). Exclusion criteria included prior treatment of tumor and inadequate imaging across CFP, ultra-widefield SLO (UWFSLO), and EDI-OCT. Lesions located in the far peripheral fundus beyond the OCT field of view were also excluded. Participants were also excluded if inadequate medical records prevented data collection.

### Study Procedures

Participants underwent ocular imaging of choroidal tumors on CFP (Canon CR6-45NM, macula-centered and lesion-centered), UWFSLO (Optos California, pseudocolor and autofluorescence [AF]), and OCT (Spectralis Heidelberg, EDI-OCT and infrared, lesion-centered). A custom software application, Cross-modality Annotation Software (XMAS),^6^ developed at the Centre for Eye Research Australia, was used to coregister images across modalities by using anatomical landmarks (optic disc, fovea, and vascular bifurcations) to ensure spatial correspondence. The Optos software was used for horizontal measurements—first, the shortest distance between tumor and optic nerve; second, the shortest distance between tumor and fovea; and third, the largest basal diameter (largest *en face* diameter of the tumor) as determined by an ocular oncologist. On Optos SLO and AF images, tumor location, presence of retinal pigment epithelium atrophy, hyperplasia or metaplasia, lipofuscin (determined using AF), and pigmentation level (classified by an ocular oncologist as nonpigmented, mixed-pigmented, or pigmented based on both CFP and AF images) were assessed. On EDI-OCT, the presence of subretinal fluid was noted. From medical records, participants’ ultrasound reports, baseline visual acuity, clinical diagnosis, and tumor staging were extracted. Key parameters from ultrasound—thickness, internal reflectivity, shape, hollowness, and largest basal diameter (only if peripherally located and measurement on Optos was not possible)—were determined from ultrasound records taken from patient medical records.

### Image Grading

All image processing was performed using the Cross-modality Annotation Software (XMAS, Centre for Eye Research Australia).^6^ Lesion-centered CFP, alongside SLO, infrared, and EDI-OCT images, were first coregistered across the different imaging modalities so that the retinal location of each pixel was overlapped for each modality.

**Figure 1:**
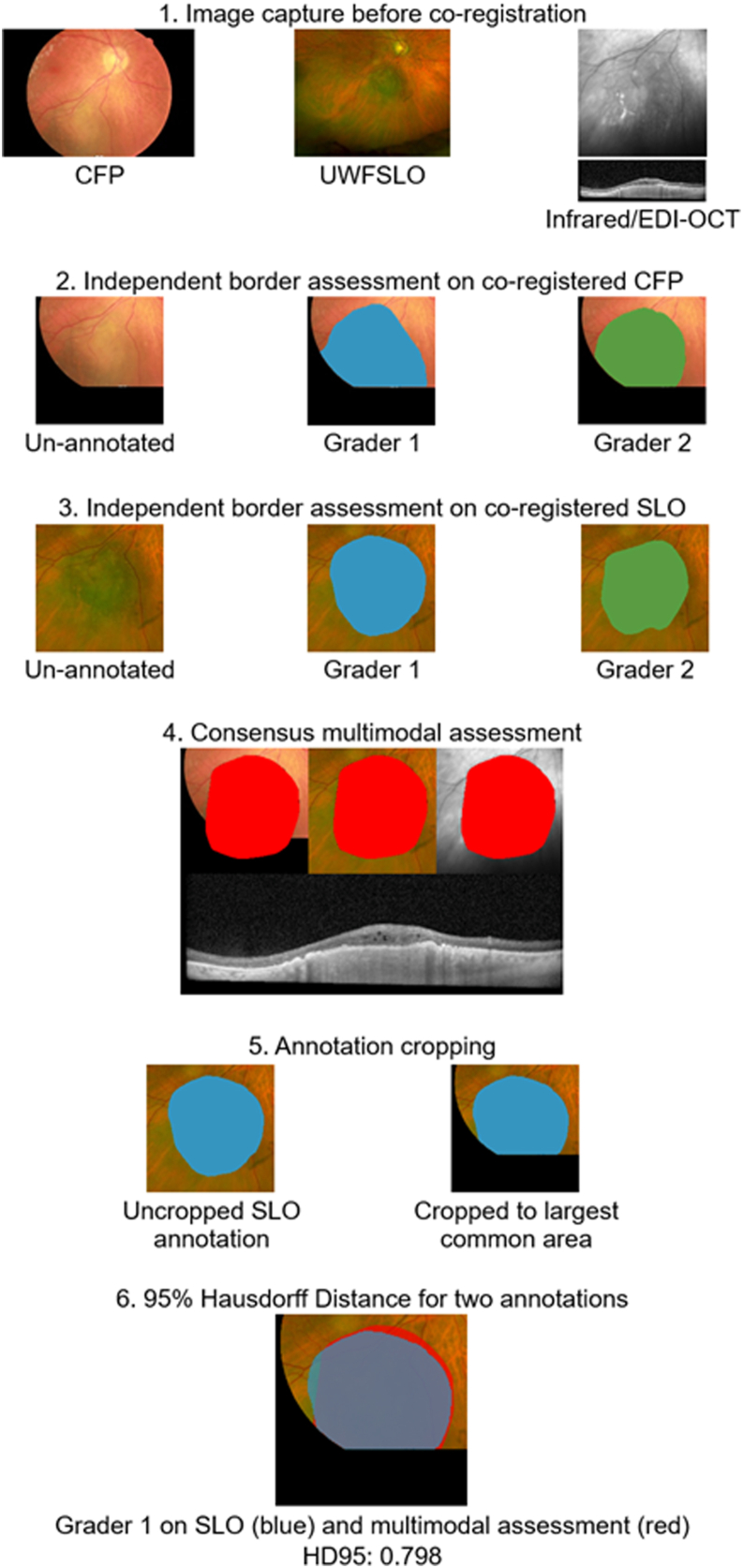
Imaging manipulation and obtaining 95% Hausdorff Distance Step 1 – Image acquisition on CFP, SLO, and OCT B-scans and *en face* IR. They were co-registered to the OCT field of view. Step 2 – Independent annotation of tumor borders using CFP alone by grader 1 (blue) and grader 2 (green). Step 3 – Independent annotation of tumor borders using SLO alone by grader 1 (blue) and grader 2 (green). Step 4 – Multimodal assessment of tumor borders using OCT, SLO, and CFP by graders in collaboration (red). Step 5 – Cropping process for annotations that exceeded the common field of view across all modalities. Step 6 – 95th-percentile Hausdorff Distance assessing the degree of overlap between cropped annotation of grader 1 on SLO (blue) against multimodal assessment (red). This example yielded an HD95 value of 0.798 mm. CFP = color fundus photography; EDI-OCT = enhanced-depth imaging-OCT; HD95 = 95th-percentile Hausdorff Distance; IR = infrared; SLO = scanning laser ophthalmoscopy; UWFSLO = ultra-widefield scanning laser ophthalmoscopy.

XMAS border annotation tool was then used to annotate the images (Fig 1). Two ocular oncologists (“grader 1” and “grader 2”) independently annotated the borders of each tumor on SLO and CFP images individually. To establish the multimodal assessment, both graders performed an annotation of the tumor borders collaboratively using the coregistered images of all available imaging modalities (CFP, SLO, infrared, fundus autofluorescence, and EDI-OCT).

**Figure 2:**
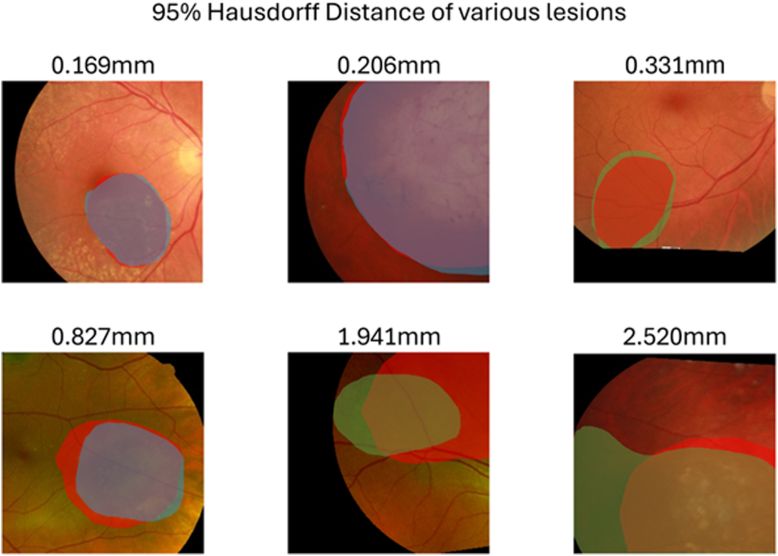
The 95% Hausdorff Distance of various lesions. The 95th-percentile Hausdorff Distances are above the respective images. Annotations and images have been cropped to show the largest common area among all imaging modalities. Top left: Grader 1 on CFP (blue) against multimodal assessment (red). Top center: Grader 1 on CFP (blue) against multimodal assessment (red). Top right: Grader 2 on CFP (green) against multimodal assessment (red). Bottom left: Grader 2 on SLO (green) against multimodal assessment (red). Bottom center: Grader 2 on CFP (green) against multimodal assessment (red). Bottom right: Grader 2 on SLO (green) against multimodal assessment (red). CFP = color fundus photography; SLO = scanning laser ophthalmoscopy.

### Quantitative Metrics

The 95th-percentile Hausdorff Distance (HD95) is a commonly used medical imaging segmentation metric that quantifies boundary agreement between 2 annotations.^7^^,^^8^ It is derived by computing the shortest distance from every boundary point on 1 annotation to the nearest point on the other. The largest of these distances is the Hausdorff Distance, and the 95th-percentile version (HD95) reduces the influence of outliers. Because this metric is expressed in millimeters, it allows direct clinical interpretation; for example, an HD95 of 2.5 mm indicates that 95% of all pointwise distances between the 2 annotations are <2.5 mm.

To ensure consistency across imaging modalities and minimize potential field-of-view bias, all annotations were cropped to the largest common region of the fundus visible across modalities, typically corresponding to the dimensions of the OCT image. This standardization provided a uniform basis for comparison. On the borders of these cropped annotations, HD95 was then calculated to enable unbiased assessment of segmentation performance.

To evaluate the accuracy of each modality and grader, HD95 values were compared between the individual annotations and the multimodal consensus assessment. The complete workflow, beginning at image acquisition and ending with HD95 computation, is summarized in Figure 1.

Values of HD95 range from 0 mm, which indicates that 95% of the 2 boundaries are almost perfectly aligned upward, with larger values signifying greater disagreement (Fig 2). We consider HD95 <2 mm to represent a clinically acceptable level of agreement. Current clinical standards recommend a 2 mm margin^9^ around a tumor border during localized treatment to account for subclinical disease. For this reason, an HD95 >2 mm is interpreted as a clinically significant variation between assessments.

We also computed the Dice coefficient, a commonly used spatial overlap index for the annotations. The summary statistics have been included in Supplementary Table 1, available at www.ophthalmologyscience.org.

### Statistical Analysis

The distribution of each characteristic was summarized as median with interquartile range for continuous variables and as frequencies with percentages for categorical variables. Because HD95 values were skewed toward 0, they were compared between graders and between imaging modalities using Wilcoxon signed-rank tests. The extent of variation in HD95 among graders and between modalities was evaluated using the median, interquartile range, and full range of HD95 values. In addition, the number of tumors with HD95 values above the clinically acceptable threshold of 2 mm was calculated.

Univariable Gaussian generalized estimating equations^10^ were employed to investigate the association between tumor or participant characteristics and the level of agreement with the multimodal assessment, as quantified by HD95. Separate models were created for each combination of grader, unimodal device, and potential predictor. Potential predictors included age, gender, eye, largest basal diameter, thickness, distance to the optic nerve and fovea, diagnosis, location of the posterior edge of the tumor, extension to the optic nerve, clock hours, pigmentation level, internal reflectivity, shape, best-corrected visual acuity (20/50 or worse), tumor staging, presence of lipofuscin, hollowness, subretinal fluid, retinal pigment epithelium atrophy, hyperplasia, metaplasia, or presence of any of these 3 retinal pigment epithelium changes.

For categorical predictors with >2 categories, the combined null hypothesis across all categories was assessed using postestimation chi-squared tests. A *P* value of ≤0.05 was considered statistically significant.

Statistical analysis was performed using Stata BE v.19 (StataCorp).

## Results

A total of 64 lesions from 63 patients were included. The median age was 63 years, and 60.9% of lesions were nevi (see Table 1).

**Table 1:** Demographics and Tumor Characteristics

Continuous: Median [Q1-Q3] Categorical: Frequency (%) n = 64	
Age at imaging (years)	63 [55–72]
Gender	
Male	35 (54.7%)
Female	29 (45.3%)
Eye	
Left	37 (57.8%)
Right	27 (42.2%)
Largest basal diameter (mm)	5.65 [3.80–9.05]
Thickness (mm)	1.85 [1.10–2.47]
Distance to nerve (mm)	3.35 [1.50–6.10]
Distance to fovea (mm)	2.85 [1.60–5.35]
Clinical diagnosis	
Choroidal nevus	39 (60.9%)
Choroidal melanoma	25 (39.1%)
Location of posterior edge of tumor	
Juxtapapillary (<1 mm from optic nerve)	12 (18.8%)
Macular	26 (40.6%)
Nasal	3 (4.7%)
Superior	8 (12.5%)
Inferior	8 (12.5%)
Temporal	7 (10.9%)
Touching optic nerve (if juxtapapillary location); n = 12	7 (58.3%)
Clock-hours (if touching optic nerve); n = 7	
1	0 (0%)
2	1 (14.3%)
3	2 (28.6%)
4	1 (14.3%)
5	3 (42.9%)
6–12	0 (0%)
Pigmentation level	
Nonpigmented	11 (17.2%)
Mixed-pigmented	12 (18.8%)
Pigmented	41 (64.1%)
Lipofuscin present	29 (45.3%)
Internal reflectivity	
Low	30 (46.9%)
Medium	16 (25.0%)
High	5 (7.8%)
Not applicable (flat)	13 (20.3%)
Shape	
Dome	51 (79.7%)
Flat	13 (20.3%)
Hollowness present	28 (43.8%)
Subretinal fluid present	33 (51.6%)
Retinal pigment epithelium atrophy present	10 (15.6%)
Retinal pigment epithelium hyperplasia/metaplasia present	9 (14.1%)
Any retinal pigment epithelium changes (atrophy/hyperplasia/metaplasia) present	15 (23.4%)
Baseline visual acuity 20/50 or worse	3 (4.7%)
Staging of cancer	
T1A	16 (25.0%)
T2A	8 (12.5%)
T3A	1 (1.6%)
Not applicable (nevus)	39 (60.9%)

### Unimodal Tumor Border Assessment on CFP and SLO (Interrater Reliability)

**Figure 3:**
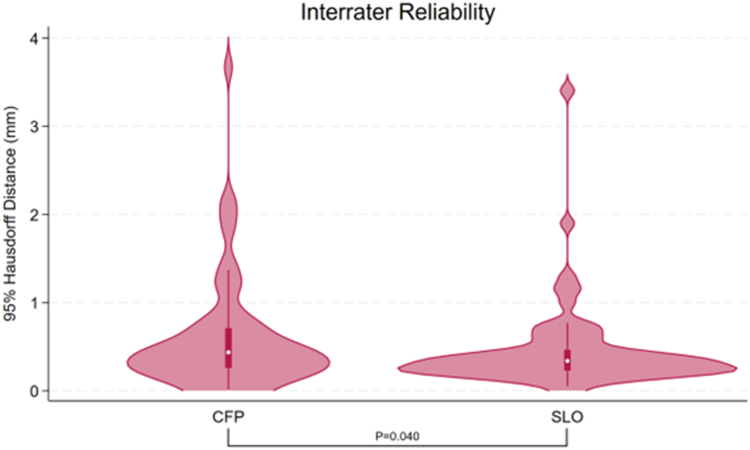
Interrater reliability of unimodal tumor border delineations on CFP and SLO. Violin plots show 95th-percentile Hausdorff Distances between 2 graders for each tumor on CFP (left) and SLO (right); overlaid boxplots indicate the median, interquartile range, and 5 to 95th percentile range. The Wilcoxon signed-rank test *P* value is for the comparison between CFP and SLO (*P* = 0.040). CFP = color fundus photography; SLO = scanning laser ophthalmoscopy.

Interrater agreement was high for CFP (median between-rater HD95 0.44 mm, interquartile range [IQR] 0.26–0.71; range 0.02–3.67), and for SLO (median HD95 0.34 mm, IQR 0.23–0.46; range 0.05–3.40). The difference in interrater agreement between modalities reached statistical significance (*P* = 0.040; see Fig 3). A between-grader HD95 ≥2 mm was observed for 3 tumors (5%) on CFP and 1 tumor (2%) on SLO.

### Comparison of CFP and SLO Tumor Border Assessment to Multimodal Assessment

**Figure 4:**
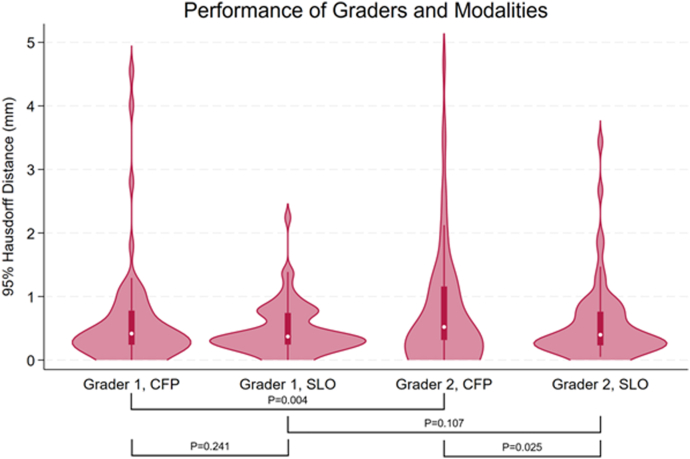
Performance of graders and modalities against multimodal assessments 95th-percentile Hausdorff Distances for tumor border delineations by 2 graders on CFP and SLO against a multimodal assessment. Violin plots show tumor assessments; overlaid boxplots indicate median, interquartile range, and 5 to 95th percentile range. Wilcoxon signed-rank test *P* values are shown for grader 1 CFP vs SLO (*P* = 0.241), grader 1 vs grader 2 on CFP (*P* = 0.004), grader 1 vs grader 2 on SLO (*P* = 0.107), and grader 2 CFP vs SLO (*P* = 0.025). CFP = color fundus photography; SLO = scanning laser ophthalmoscopy.

Unimodal tumor border delineations on CFP and SLO by 2 graders were each compared with a multimodal assessment (Fig 4). Grader 1 achieved a median between-modality HD95 of 0.42 mm (IQR 0.24–0.78; range 0–4.55) on CFP and a median of 0.37 mm (IQR 0.24–0.74; range 0–2.24) on SLO. Grader 2 recorded a median HD95 of 0.52 mm on CFP (IQR 0.31–1.16; range 0–4.68) and 0.40 mm on SLO (IQR 0.23–0.76; range 0.05–3.43). A statistically significant difference was observed between graders on CFP (*P* = 0.004), but not for SLO (*P* = 0.107). Between-modality agreement was higher for SLO than CFP for grader 2 (*P* = 0.025), but not for grader 1 (*P* = 0.241).

Across all tumors, HD95 was >2 mm for 5% of cases assessed by grader 1 on CFP and 2% on SLO, and for 9% of cases assessed by grader 2 on CFP and 3% on SLO.

### Tumor Characteristics Affecting Delineation on Unimodal or Multimodal Assessment

**Figure 5:**
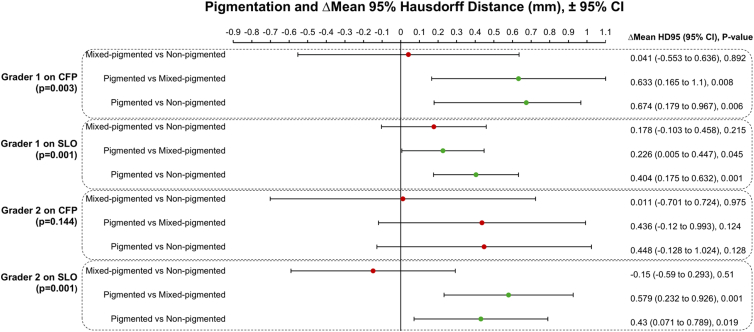
Mean differences in 95th-percentile Hausdorff Distances for agreement with the multimodal assessment (Δmean HD95) between tumor pigmentation categories, stratified by grader and device, derived from generalized estimating equations. CFP = color fundus photography; CI = confidence interval; HD95 = 95th-percentile Hausdorff Distance; SLO = scanning laser ophthalmoscopy.

Among all lesion characteristics tested, pigmentation significantly influenced between-modality agreement for 3 of the 4 grader-modality pairs (overall *P* < 0.05; Fig 5). Statistical significance was not reached for grader 2 on SLO. No other characteristic, including horizontal tumor size, demonstrated a statistically significant effect.

Between-modality HD95 values were higher, indicating greater disagreement, on average, for nonpigmented tumors than for pigmented tumors for grader 1 on CFP (Δmean HD95 = 0.674, *P* = 0.006) and SLO (Δmean HD95 = 0.404, *P* = 0.001), and also for grader 2 on SLO (Δmean HD95 = 0.43, *P* = 0.019). Between-modality HD95 values were also higher for mixed-pigmented tumors than for pigmented tumors for the same grader-modality pairs. No statistically significant differences in HD95 were observed between mixed-pigmented and nonpigmented tumors for any grader-modality pair.

Suboptimal agreement with the multimodal assessment (HD95 ≥2 mm) was observed for pigmented tumors in 0% (0 of 41) of grader 1 CFP and SLO assessments and in 5% (2 of 41) and 0% (0 of 41) of grader 2 CFP and SLO assessments, respectively. For mixed-pigmented lesions, HD95 ≥2 mm occurred in 17% (2 of 12) of grader 1 CFP assessments, 8% (1 of 12) of grader 1 SLO assessments, 25% (3 of 12) of grader 2 CFP assessments, and 17% (2 of 12) of grader 2 SLO assessments. For nonpigmented tumors, HD95 ≥2 mm occurred in 9% (1 of 11) of assessments by grader 1 on CFP, 0% (0 of 11) by grader 1 on SLO, 9% (1 of 11) by grader 2 on CFP, and 0% (0 of 11) by grader 2 on SLO.

## Discussion

Our study highlights 2 key findings. First, there is measurable intergrader variability in choroidal tumor border delineation, even among experienced ocular oncologists. Second, a significant subset of tumors, particularly nonpigmented or mixed-pigmented lesions, were inaccurately delineated when assessed with CFP or SLO alone. These findings underscore the limitations of relying solely on unimodal *en face* imaging and emphasize the need for multimodal assessment protocols in ocular oncology.

While quantitative assessment of choroidal tumor borders has been explored in related radiologic and radiotherapy imaging studies,^11^ systematic evaluation using ophthalmic multimodal imaging remains limited. Prior studies largely emphasized tumor thickness, basal diameter, or qualitative features rather than precise border mapping. In our cohort, overall 95% of Hausdorff Distances were low (≤2 mm), but nearly 7% of CFP-based and 2% of SLO-based delineations fell below the clinically acceptable threshold. This indicates that variability depends not only on imaging modality but also on grader interpretation, particularly for lesions with reduced pigmentation or indistinct borders.

Color fundus photography and SLO remain the mainstay of choroidal tumor documentation because of their availability and widefield of view, yet both have notable limitations. Color fundus photography relies on pigment contrast and illumination, which are often insufficient in hypopigmented or peripheral lesions. Scanning laser ophthalmoscopy generally produces sharper contrast and higher agreement but still fails in nonpigmented or mixed-pigmented tumors. These observations align with prior reports showing that *en face* imaging may underestimate lesion margins or fail to capture posterior extent.^12^ Other variables, including tumor basal diameter, distance to nerve or fovea, thickness as measured on ultrasound, or other clinical characteristics, were not correlated with greater or lower agreement in border delineation, despite their importance in other aspects of tumor assessment and management.

The literature on intergrader reliability in choroidal tumor border delineation is sparse. Paul Brett et al^12^ compared UWFSLO and CFP for nevus analysis using a questionnaire, while Wang et al,^13^ Martins et al,^14^ Lee et al,^15^ and Torres et al^16^ focused on thickness or diameter rather than margins. Machine learning has also been explored; Ma et al^17^ developed an algorithm to map tumor borders from UWFSLO, but training relied on a single clinician’s annotations, and border accuracy was not validated. Other modality comparisons highlight similar limitations. Baradad-Jurjo et al^18^ reported good concordance between ultrasonography and ultra-widefield fundus imaging for basal diameter measurements, but more than half of patients were excluded because of indistinct borders. Similarly, Muftuoglu et al^19^ found that multicolor imaging could capture lesion features comparable to CFP but underestimated lesion size by about 33%. Together, these studies provide important groundwork but do not directly evaluate reproducibility or accuracy of margin delineation, underscoring the novelty of our focus on precise border mapping.

OCT-based techniques have broadened lesion characterization beyond surface appearance and show particular promise for boundary assessment. Swept-source OCT provides detailed visualization of intralesional structures, including vasculature and abnormal choriocapillaris, features less apparent with EDI-OCT. Francis et al^20^ demonstrated that swept-source OCT can reliably visualize the choroidoscleral boundary and suprachoroidal space, while Jonna and Daniels^5^ identified subtle elevation patterns in flat nevi using EDI-OCT, classifying 5 morphologic subtypes with clinical relevance. Novel approaches such as dark-field SLO have also shown high sensitivity and reproducibility in delineating lesion borders, outperforming *en face* OCT and CFP in assessing border sharpness and pigmentation contrast.^21^ Such developments highlight the potential for new technologies to overcome the current limitations of *en face* imaging in border mapping.

In our study, SLO slightly outperformed CFP for unimodal mapping, but neither modality reliably captured hypopigmented lesions, where 95% Hausdorff Distances ≥2 mm occurred in up to 9% of nonpigmented tumors and up to 25% of mixed-pigmented tumors. Incorporating OCT, particularly EDI-OCT, added valuable cross-sectional information that clarified margins when pigment contrast was poor. The combination of modalities therefore provided the most reliable assessment, consistent with prior evidence that OCT features are critical for diagnosis and monitoring of melanocytic lesions. Limitations remain, particularly in peripheral lesions where OCT coverage is constrained. Although ultrasound data were available for most participants, direct comparison of ultrasound-derived margins with OCT and CFP was not feasible because ultrasound images were not spatially co-registered. However, ultrasound remained useful for assessing tumor thickness and basal diameter in peripheral lesions where *en face* imaging was incomplete.

These findings have several implications for clinical practice. Clinicians should recognize the limitations of CFP and SLO when assessing nonpigmented or mixed-pigmented lesions and should incorporate OCT for margin determination whenever borders are indistinct. This is particularly relevant when planning radiotherapy for posterior or partially pigmented melanomas, where failure to encompass the true tumor extent may increase the risk of local recurrence. Our results also raise awareness that tumor margin delineation is not always reproducible, even among experts, and that complex cases may warrant review in subspecialty centers rather than community practice. Finally, they highlight a gap in available imaging technology, with a clear need for *en face* modalities such as widefield OCT or hyperspectral imaging^22^ that could provide more accurate and objective border mapping.

Strengths of our study include a diverse cohort of nevi and melanomas, systematic image coregistration across modalities, and consensus-based multimodal delineations performed by subspecialist ocular oncologists. Our sample size also exceeds prior reports, allowing subgroup analyses by pigmentation and morphology. Limitations include the predominance of nevi, which may reduce generalizability to larger melanomas, occasional truncation of peripheral lesions on CFP, and the inherent subjectivity of manual grading. Tumors located in the periphery may be imaged with greater deformity; however, this was standardized across all modalities for peripherally located tumors with the use of coregistration software. Lesions too peripherally located to be imaged clearly on OCT were excluded. Pigmentation was assessed using both color fundus photographs and AF images; however, clinical appearance may differ from these imaging modalities due to lighting, media opacity, and camera calibration, which could have introduced minor bias in categorizing pigmentation levels.

Future research should focus on advanced imaging modalities that extend beyond current clinical practice. While OCT is already standard for lesion evaluation, emerging techniques such as widefield *en face* OCT and hyperspectral imaging^22^ may overcome the limitations of CFP and SLO in defining margins. Machine learning approaches also hold promise, but as our findings demonstrate, the variability in clinician-determined borders highlights the need for large, multimodal training datasets before clinical application is feasible. Prospective studies should further evaluate whether integrating OCT-based and next-generation border mapping tools into clinical protocols reduces recurrence risk or improves treatment outcomes.

## Conclusion

Our study demonstrates that a subset of choroidal tumors, particularly nonpigmented and mixed-pigmented lesions, are inaccurately delineated with unimodal imaging alone. Multimodal imaging, including EDI-OCT, is critical for precise tumor border delineation, guiding treatment planning, and improving clinical outcomes. Awareness of these limitations should inform clinical practice, and future advances in imaging technology and artificial intelligence may further enhance the accuracy and consistency of tumor margin assessment.
