# Supplementary material for: Discrepancies between Fundus Photography and Multimodal Imaging in Mapping of Choroidal Tumor Borders
Source: Ophthalmol Sci. 2025 Dec 30;6(3):101057. doi: 10.1016/j.xops.2025.101057 (PMC12907106; doi:10.1016/j.xops.2025.101057)
Supplement: Supplementary Table 1 [file mmc1.docx]

Supplementary Table 1: Dice Coefficient Summary Statistics

|  | Dice Coefficient  Median (interquartile range) [range] |
| --- | --- |
| Grader 1 on CFP against Grader 2 on CFP | 0.925 (0.89-0.951), [0.667-0.999] |
| Grader 1 on SLO against Grader 2 on SLO | 0.943 (0.916-0.967), [0.564-0.998] |
| Grader 1 on CFP against multimodal assessment | 0.932 (0.894-0.955), [0.276-1] |
| Grader 1 on SLO against multimodal assessment | 0.93 (0.911-0.964), [0.71-1] |
| Grader 2 on CFP against multimodal assessment | 0.901 (0.824-0.944), [0.278-0.999] |
| Grader 2 on SLO against multimodal assessment | 0.926 (0.882-0.957), [0.592-0.998] |
